# Clinical Presentation and Treatment of Thromboangiitis Obliterans With Superimposed Osteomyelitis

**DOI:** 10.7759/cureus.21423

**Published:** 2022-01-19

**Authors:** Ariel Kidron, Christian Daahir

**Affiliations:** 1 Emergency Medicine, Nova Southeastern University Kiran C. Patel College of Osteopathic Medicine, Fort Lauderdale, USA; 2 Anesthesiology, Aventura Hospital and Medical Center, Aventura, USA

**Keywords:** derm-rheum, buerger’s disease, re-vascularization, cutaneous vasculitis, small vessel vasculitis

## Abstract

Thromboangiitis obliterans (TAO) is an autoimmune vasculitis that typically presents in male smokers between the ages of 25 and 40. Although TAO primarily affects medium-sized blood vessels, it can also infiltrate small blood vessels as well as nerves and veins. The inflammation can cause segmental thrombosing and occlusion of the blood vessels leading to gangrene and eventual amputations. This case presents a unique sequela of TAO, in which a 28-year-old male with a chronic tobacco smoking history was diagnosed with TAO after presenting with pain and discoloration in his foot, in addition to superimposed osteomyelitis. This case serves to remind emergency clinicians, internal medicine physicians, general practitioners, and infectious disease specialists of the TAO differential in patients presenting with pain and discoloration in the feet, as well as the importance of working up the patient for any superimposed infections.

## Introduction

Thromboangiitis obliterans (TAO), otherwise known as Buerger’s disease, is a rare, locally aggressive, vascular occlusive disease of the small and medium vessels that is highly associated with smoking and tobacco usage. Among patients with peripheral vascular disease, the prevalence and incidence of TAO have been estimated at 0.75% and 8-12.6 cases per 100,000 people, respectively [1]. TAO typically appears on the extremities and can be characterized by concurrent invasion of the nerves and veins potentially leading to gangrene [2]. Here, we report a case of the clinical and histopathological presentation of TAO along with the medical and surgical treatment modality adopted to treat the condition.

## Case presentation

**Figure 1:**
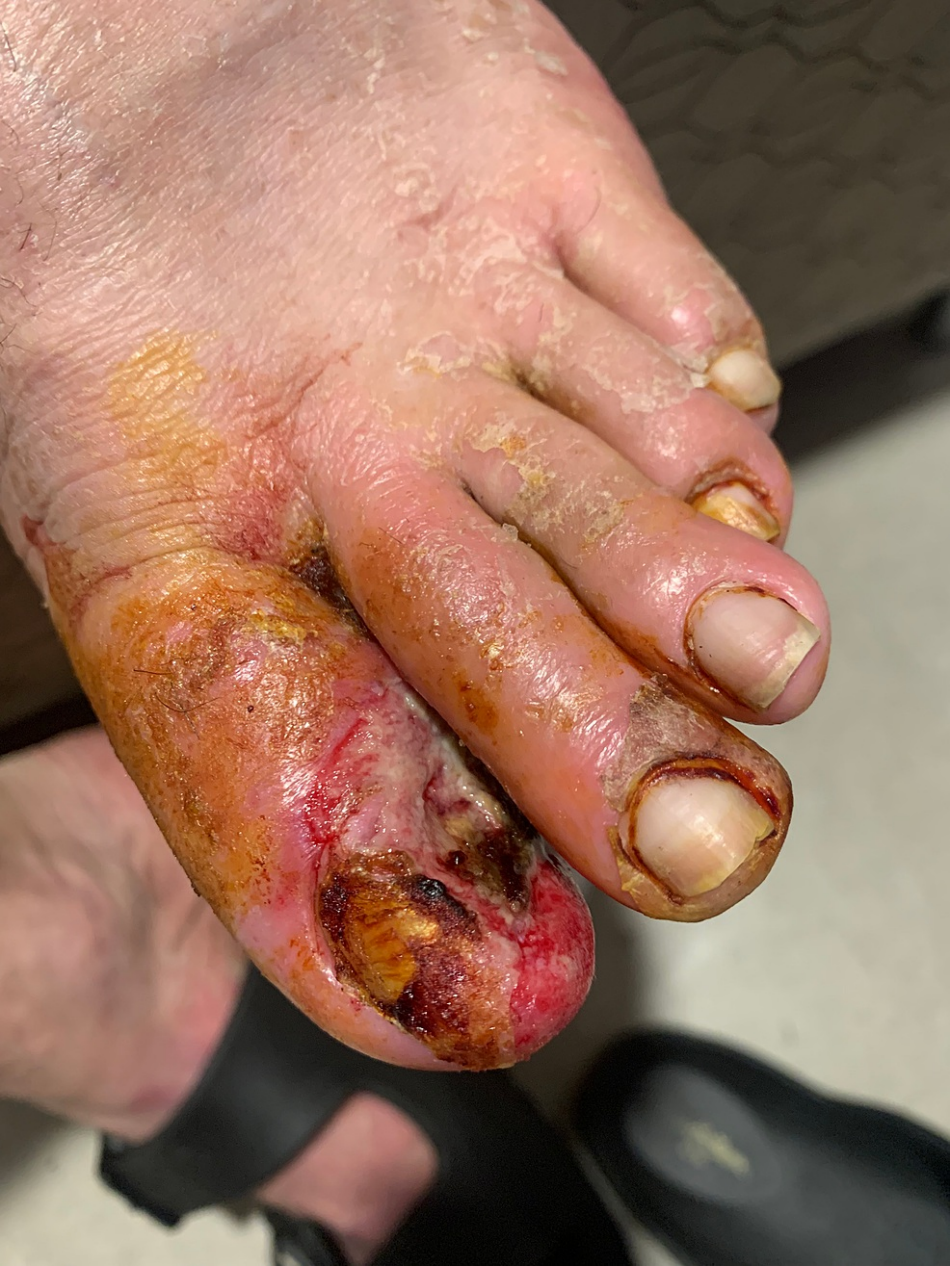
Initial presenting lesion on the left foot showing tissue necrosis and exposed bone on the hallux.

A 28-year-old male, chronic smoker with a 10 pack-year history, as well as a medical history of osteomyelitis of the left hallux treated with intravenous (IV) antibiotics, presented to the emergency department with 5/10 sharp pain in his left hallux that increased upon laying down with no improvement. The patient reported slight chills but with normal vitals, laboratory values, and blood cultures. Physical examination of the left hallux revealed local swelling, redness, and exposed bone with non-palpable dorsalis pedis and posterior tibial pulses (Figure 1).

**Figure 2:**
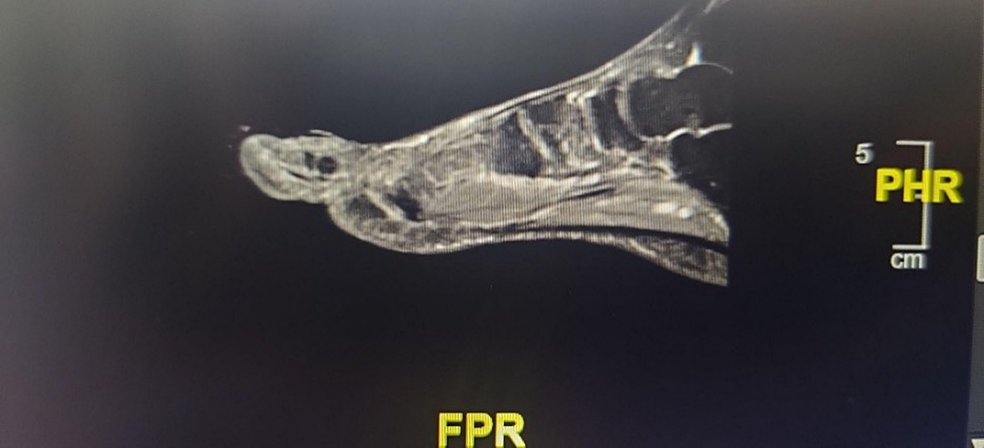
T2-weighted MRI showing soft tissue swelling and ulceration along with near exposure of the bone characteristic for osteomyelitis. MRI: magnetic resonance imaging

An initial plain radiograph of the left foot showed a periosteal reaction consistent with osteomyelitis to the distal phalanx of the left hallux. A bilateral lower extremity arterial study showed concordant pulse volume recording waveforms. This technique is used to calculate blood volume changes in legs that are displayed as a waveform. The patient displayed minimal changes in volume signifying occlusion to blood flow. He then had a bilateral lower extremity angiogram that showed dampened monophasic flow in the lower legs bilaterally with occlusion of all three tibial arteries at the level of the mid-calf with multiple corkscrew collaterals extending toward the left foot. Monophasic flow signified that the arteries being tested displayed only a forward systolic flow continuing into diastole and lacked the reverse diastolic flow characterized in normal arteries. Thus, the diagnosis of TAO was confirmed. A left foot magnetic resonance imaging showed soft tissue swelling and ulceration along the lateral aspect of the great distal toe with near exposure of the bone characteristic for osteomyelitis (Figure 2).

Deep wound cultures and bone pathology were positive for infection with pansensitive *Pseudomonas aeruginosa*. The patient was subsequently placed on a four to six-week course of IV 2 g of cefepime twice a day. In addition, the patient underwent surgical soft tissue debridement of the left foot and was successfully treated with recanalization and angioplasty of the anterior tibial artery and dorsalis pedis artery with a resultant single vessel runoff to the foot and toes. Patency of the vessels was supported with nitroglycerin patches, 81 mg aspirin, and 75 mg clopidogrel once daily, along with adaptive dry sterile dressing until wound healing. The patient was placed on Tylenol, ketorolac, and Percocet as needed for pain management. In addition, he was counseled on smoking cessation and the relevant association between tobacco use and TAO. On follow-up at three weeks, no recurrence was observed. The patient was able to ambulate without complaints; however, he maintained his smoking habits and was further counseled on smoking cessation.

## Discussion

The patient’s left foot vessels were vascularly consistent and clinically typical of TAO. In TAO, imaging typically reveals proximal vasculature sparing, segmental occlusion, frequent involvement of anterior and posterior tibial arteries, and development of collaterals [3]. It typically presents with intermittent claudication and painful ulceration often leading to autoamputation of digits due to the involvement of nerves and vessels predominantly in heavy male smokers most commonly between the ages of 25 and 50 [4]. According to the literature, the pathophysiology is hypothesized to be a combination of immunologic hypersensitivity to tobacco extracts, defects in endothelium-dependent vasodilation, and anti-endothelial cell antibodies [4]. This case is consistent with the manifestations of vasculitis; however, the case was particularly unique as the patient had concurrent osteomyelitis superimposed on the inflammation which further complicated the management and prognosis of the underlying condition.

The only unanimously recognized treatment is smoking cessation. In a prospective cohort study of 89 patients followed for 91.6 months, amputation rates were 42% in patients who continued to smoke as opposed to 5% in those who stopped smoking [5]. Multiple clinical trials have reported improvements in long-term benefits and outcomes by maximizing and maintaining vessel patency [6]. Thus, due to complete multiple vessel occlusion in our patient, our team adopted a bimodal approach toward treatment, including a simultaneous surgical recanalization of the tibial vessels as well as vasodilators and anticoagulation agents [7].

TAO has a similar presentation to systemic vasculitis that includes painful skin ulcerations, myalgias, and Raynaud’s phenomenon. Systemic vasculitis can have renal, pulmonary, and cardiovascular complications which are typically lacking in TAO. Systemic vasculitis is commonly treated with immunosuppressive and steroidal medications such as cyclophosphamide, methotrexate, and prednisone; however, immunosuppressive therapy has shown no clear benefit in TAO [7]. Furthermore, because our patient had concurrent osteomyelitis, immunosuppressive therapy could have proven to be detrimental as it can lower the effective lymphocyte count and hinder patients’ immune systems from being able to mount an effective defense against the infection. Treatment for osteomyelitis caused by *Pseudomonas aeruginosa* may entail a four to six-week IV regimen of either ceftazidime or cefepime combined with an aminoglycoside or piperacillin-tazobactam depending on bacterial sensitivity and clinical correlation [8].

## Conclusions

Given the complexity of the diagnostic considerations and therapeutic approaches, it is crucial for clinical practitioners to recognize the acute presentation and management of TAO and initiate a multidisciplinary approach with early vascular surgery consultation to provide balanced and multifaceted care.
